# Long-term risk-factor control and secondary prevention are insufficient after first TIA: Results from QregPV

**DOI:** 10.1177/23969873231215629

**Published:** 2023-11-30

**Authors:** Johan-Emil Bager, Katarina Jood, Annika Nordanstig, Tobias Andersson, Jonatan Nåtman, Per Hjerpe, Annika Rosengren, Georgios Mourtzinis

**Affiliations:** 1 Department of Molecular and Clinical Medicine, Institute of Medicine, Sahlgrenska Academy, University of Gothenburg, Gothenburg, Sweden; 2 Department of Emergency Medicine, Sahlgrenska University Hospital, Gothenburg, Sweden; 3 Department of Clinical Neuroscience, Institute of Neurosciences and Physiology, Sahlgrenska Academy, University of Gothenburg, Gothenburg, Sweden; 4 Department of Neurology, Sahlgrenska University Hospital, Gothenburg, Sweden; 5 Primary Health Care, School of Public Health and Community Medicine, Institute of Medicine, Sahlgrenska Academy, University of Gothenburg, Gothenburg, Sweden; 6 Regionhälsan R&D Centre, Skaraborg Primary Care, Skövde, Sweden; 7 Centre of Registers Västra Götaland, Gothenburg, Sweden; 8 Sahlgrenska University Hospital/Östra, Gothenburg, Sweden; 9 Department of Medicine and Emergency Mölndal, Sahlgrenska University Hospital, Gothenburg, Sweden

**Keywords:** Transient ischemic attack, cerebrovascular disease, hypertension, low-density lipoprotein cholesterol, smoking, primary health care

## Abstract

**Introduction:**

Long-term risk-factor control and secondary prevention are not well characterized in patients with a first transient ischemic attack (TIA). With baseline levels as reference, we compared primary-care data on blood pressure (BP), low-density lipoprotein cholesterol (LDL-C), smoking, and use of antihypertensives, statins and antiplatelet treatment/oral anticoagulation (APT/OAC) during 5 years after a first TIA.

**Patients and methods:**

Patients in QregPV, a Swedish primary-care register for the Region of Västra Götaland, with a first TIA discharge diagnosis from wards proficient in stroke care 2010 to 2012 were identified and followed up to 5 years. BP, LDL-C, smoking, use of antihypertensives, statins, APT/OAC, and achievement of target levels were calculated. We used logistic mixed-effect models to analyze the effect of follow-up over time on risk-factor control and secondary prevention treatment.

**Results:**

We identified 942 patients without prior cerebrovascular disease who had a first TIA. Compared to baseline, the first year of follow-up was associated with improvements in concomitant attainment of BP <140/90 mmHg, LDL-C < 2.6 mmol/L and non-smoking, which rose from 20% to 33% (OR 2.08, 95% CI 1.38–3.13), but then stagnated in years 2–5. In the first year of follow-up, 47% of patients had complete secondary prevention treatment (antihypertensives, APT/OAC and statin), but continued follow-up was associated with a yearly decrease in secondary prevention treatment (OR 0.94, 95% CI 0.94–0.98).

**Conclusion:**

Risk-factor control was inadequate, leaving considerable potential for improved secondary prevention treatment after a first TIA in Swedish patients followed up to 5 years.

## Graphical abstract

**Graphical abstract:**
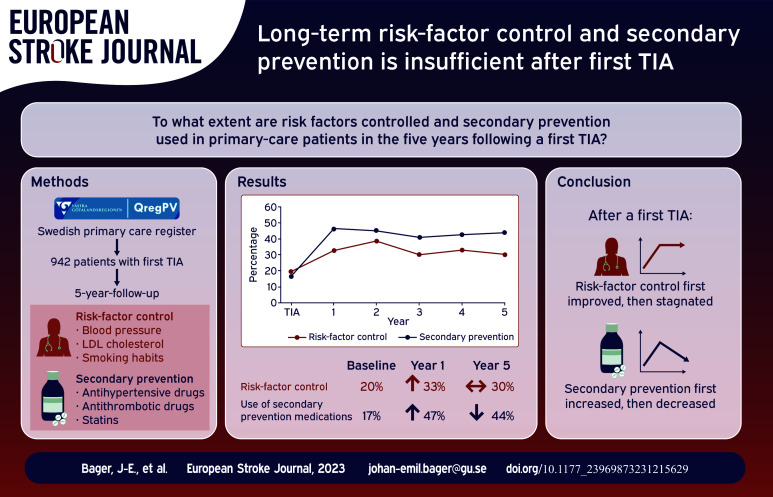

## Introduction

After a transient ischemic attack (TIA) about 10% of patients suffer a stroke within 5 years.^1^ Stroke is the second leading cause of death and disability in the world.^2^ The annual cost of stroke was estimated at 60 billion euro annually in 32 European countries in 2017.^3^ Hypertension, smoking and dyslipidemia are major modifiable risk factors for ischemic stroke and important to address after a TIA.^4–6^ Smoking cessation, adequate secondary prevention with antiplatelet therapy (APT) or oral anticoagulants (OAC), antihypertensives and statins to control blood pressure (BP) and LDL cholesterol can more than halve the risk of stroke.^7^

In Sweden, about 10,000 of 8.3 million adults are estimated to experience a TIA every year.^8^ In the Swedish region of Västra Götaland, patients with TIA are generally offered one hospital-based, outpatient follow-up visit 3 months after hospital discharge and are then referred to primary care for follow-up and risk factor-control. Despite their importance for risk of stroke, little is known about long-term risk-factor control and secondary prevention treatment after a first TIA. Several studies have featured patients with TIA, but none of them present long-term, longitudinal comprehensive data on secondary prevention treatment use and risk factor control.^9–11^

To add knowledge on long-term management after a first TIA, we used primary care data to describe 5-year trends of blood pressure control, low-density lipoprotein cholesterol (LDL-C) control and smoking habits in patients in the Region of Västra Götaland, Sweden. We also aimed to determine the use of secondary prevention treatment – antihypertensives, statins and antiplatelet treatment/oral anticoagulation (APT/OAC) – in this population.

## Patients and methods

### Data sources

The main data source for this longitudinal, observational study was QregPV, a primary-care quality of care register for the Region of Västra Götaland in Sweden which was initiated in 2010. QregPV comprises data from all primary-care patients in the Region of Västra Götaland with a diagnosis of hypertension, ischemic heart disease, diabetes mellitus, chronic obstructive pulmonary disease or asthma. In 2017, QregPV had data on 392 277 patients with these diagnoses. The register collects data from routine clinical practice and holds information on blood pressure, low-density lipoprotein cholesterol and smoking habits from all primary care centers in the Region of Västra Götaland (population 1.67 million in 2017). For this study, data from QregPV was linked to data on mortality, diagnoses and drug dispensation from national and regional registries through the personal identification number unique to every Swedish resident. QregPV and this linkage process has been described in detail elsewhere.^12^

### Study population

**Figure 1.**
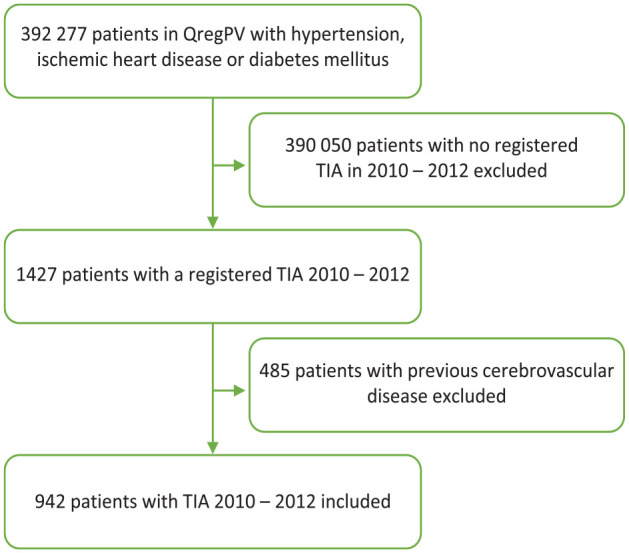
Flow chart of patient inclusion from a primary-care quality of care register, QregPV, to the study population.

Among patients registered in QregPV, we identified all patients with a principal diagnostic code of TIA (International Statistical Classification of Diseases Tenth Revision (ICD-10) codes G45.0, G45.1, G45.3, and G45.9) during a 3-year period 2010 through 2012 registered at discharge from stroke units, and wards specialized in internal medicine, neurology or geriatrics. We only included discharge diagnoses from these disciplines, which in Sweden all frequently encounter and manage patients with TIA, to increase the specificity of the TIA diagnosis, which is lower than that of stroke diagnoses.^13,14^ The day of discharge with a TIA diagnosis was defined as the index date. Patients with previous cerebrovascular disease (defined as the presence of any of the ICD-10 codes G45.0, G45.1, G45.3, G45.9, I61, I63 [except I63.6], I64 or I69) were excluded, see Figure 1. Patients were followed for 5 years after the index date, until death, or until the end of follow-up on December 31, 2017, whichever occurred first. Year 1 of follow-up was defined as the first 365 days after the index date, year 2 as day 366–730 after the index date and so on.

### Variables

Data on BP, LDL-C, smoking, glycated hemoglobin A1c (HbA1c) and body-mass index (BMI) were obtained from QregPV. For baseline variables, the last measurement prior to the index date was used. During follow-up, when multiple registrations of a variable were available for any single year, the mean of the values was used. For smoking, the last observation carried forward was used to impute missing data. Data on mortality, comorbidities, and drug treatment were obtained from national and regional registers. Baseline comorbidities were defined as any registration in these registries of the following ICD-10 diagnoses prior to the index date: hypertension (I10–I15); ischemic heart disease (I20–I25); diabetes mellitus (E10–E11); heart failure (I50); atrial fibrillation (I48); chronic obstructive pulmonary disease (J44); and peripheral arterial disease (I70, I74). We used the Anatomical Therapeutic Chemical (ATC) Classification System to define drug classes. For this study, antihypertensive treatment was defined as any registration of the following ATC codes: renin-angiotensin-aldosterone-system blockers (C09A, C09B, C09C, C09D); calcium-channel blockers (CCBs) (C07FB02, C08CA, C09BB, C09DB); beta-blockers (C07AA, C07AB, C07AG); loop diuretics (C03C); potassium-sparing diuretics (C03D, C03EA); thiazide diuretics (C03A, C03B, C03EA, C09BA, C09DA); and other antihypertensives (C02CA04, C02AC05). Antiplatelet therapy (APT) was defined as the drug classes acetylsalicylic acid (B01AC06); P2Y_12_ inhibitors (B01AC04, B01AC22, B01AC24); and dipyridamole (B01AC07). Oral anticoagulant therapy (OAC) was defined as warfarin, dabigatran, rivaroxaban, apixaban and edoxaban (B01AA03, B01AE07, B01AF01, B01AF02 and B01AF03). Statin treatment was defined as both the statins (C10AA, C10BA) and ezetimibe (C10AX09, C10BA02, C10BA05, C10BA06). Drug class use was defined, through drug dispensing data, as a medical possession ratio (MPR) of ⩾80% in the year preceding the index date and per year of follow-up after the index date.^15^ In this study, patients with concomitant BP <140/90 mmHg and LDL-C < 2.6 mmol/L (equivalent to ~100 mg/dL) who were also non-smokers were considered to have all those three risk factors controlled, based on the guideline-mandated targets that were applicable during the study.^6,16,17^ Complete secondary prevention treatment was defined as concomitant use of antihypertensives and APT/OAC and statins.

### Statistical analysis

Continuous variables are presented as mean with standard deviation, whereas categorical variables are presented as absolute and relative frequencies. We calculated and plotted risk-factor control and secondary prevention treatment with 95% confidence intervals (95% CI) per year of follow-up. Terminal digit preference was examined through calculation of the prevalence of both BP <140/90 and BP ⩽140/90 mmHg.^12,18^ When calculating yearly prevalence of complete risk-factor control, only patients with available data on all three risk factors – BP and LDL-C and smoking – were included. To analyze the effect of follow-up over time on risk-factor control and secondary prevention treatment, we used logistic mixed-effect models from the Generalized Estimation Equation (gee) package for R, which took into account repeated measurements from the same patients by including their identification numbers as a random-effect variable. Risk-factor control and prevalence of secondary preventive treatments were the dependent variables in these models and year of follow-up was the independent variable. We analyzed both change from the baseline year to year 1 of follow-up and change from year 1 to year 5 of follow-up, to differentiate the immediate effect of the TIA on risk-factor control and secondary prevention treatment in the first year of follow-up, from that of follow-up from year 1 through 5. The analyses were adjusted for age and sex. A *p* value <0.05 was considered statistically significant. All analyses were performed in R (version 4.3.1) through RStudio (version 2023.06.01, build 524).^19,20^

## Results

We identified 942 patients in QregPV free of previous cerebrovascular disease who had a hospital discharge diagnosis of TIA between 2010 and 2012. At baseline, the mean age was 75 years and women comprised 53.5% of the study population, see Table 1. Hypertension was the most common comorbidity (84.3%), followed by diabetes mellitus (37%) and ischemic heart disease (31%). Prior to the TIA, 47% of patients had a blood pressure <140/90 mmHg; 40% attained LDL-C < 2.6 mmol/L; and 15% were smokers. Antihypertensive treatment was used by 72%; APT/OAC by 41%; and statins by 31% of patients.

**Table 1.** Baseline characteristics retrieved from QregPV of 942 patients prior to first TIA in 2010–2012.

Characteristic		Missing data *n* (%)
Age, years, (SD)	75.0 (10.3)	
Female sex, *n* (%)	504 (53.5%)	
Systolic blood pressure, mmHg (SD)	138.8 (18.9)	116 (12.3%)
Diastolic blood pressure, mmHg (SD)	78.1 (12.4)	116 (12.3%)
Blood pressure <140/90 mmHg, *n* (%)	393 (47.6)	116 (12.3%)
LDL cholesterol, mmol/L (SD)	3.0 (1.1)	484 (51.4%)
LDL cholesterol < 2.6 mmol/L, *n* (%)	180 (39.3)	484 (51.4%)
HbA1C, mmol/mol (SD)	52.6 (12.7)	683 (72.5%),*
Body-mass index (SD)	28.1 (4.7)	467 (49.6%)
Smoker, *n* (%)	69 (15.0%)	482 (51.2%)
Hypertension, *n* (%)	794 (84.3%)	
Ischemic heart disease, *n* (%)	295 (31.3%)	
Diabetes mellitus, *n* (%)	349 (37%)	
Heart failure, *n* (%)	181 (19.2%)	
Atrial fibrillation, *n* (%)	170 (18%)	
Chronic obstructive pulmonary disease, *n* (%)	125 (13.3%)	
Peripheral arterial disease, *n* (%)	40 (4.2%)	

SD: standard deviation; LDL: low density lipoprotein; HbA1c: glycated hemoglobin; QregPV: quality register for primary care (“PrimärVård” in Swedish) in the Region of Västra Götaland, Sweden.

* Among 349 patients with a diagnosis of diabetes mellitus, HbA1C was missing for 81 (23.2%).

### Risk-factor control

**Figure 2.**
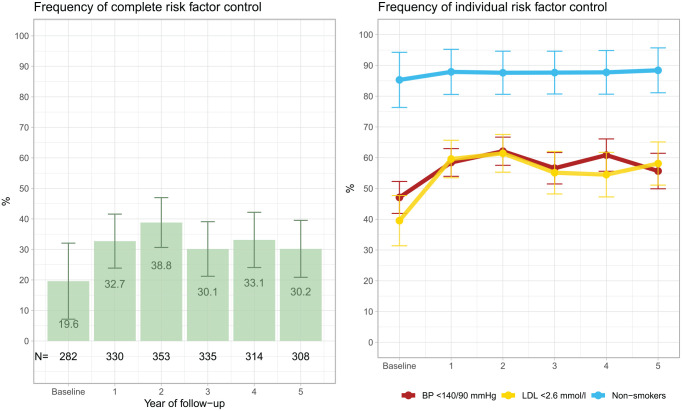
Relative frequency of attainment of target levels of all risk factors (left) and of individual risk factors (right) after a first transient ischemic attack. The black numbers at the bottom of the left plot show the absolute number (N) of patients for whom complete risk-factor data were available. BP denotes blood pressure and LDL denotes low-density lipoprotein cholesterol.

In total, 247 patients died within 5 years of their TIA, leaving 695 patients at the end of follow-up, see Table 2. One patient died on the day of the TIA diagnosis and thus did not have any follow-up. The relative frequency of blood pressure <140/90 mmHg and LDL-C < 2.6 mmol/L improved from 48% and 39% to 58% and 60%, respectively, during the first year (Figure 2). When blood pressure ⩽140/90 mmHg was instead defined as controlled, 71% of the patients reached the target in year 1 of follow-up. Smoking prevalence decreased from 15% to 12%. Complete risk-factor control, that is, the concomitant attainment of BP <140/90 mmHg, LDL-C < 2.6 mmol/L and non-smoking, was present in 20% of patients at baseline. During follow-up, complete risk-factor control improved numerically and was highest in the second year of follow-up after TIA, 39%, (Figure 2). Yearly data on all three risk factors was only available for less than 40% of the patients, however.

**Table 2.** Follow-up characteristics after first transient ischemic attack.

	Year of follow-up after first TIA				
	1	2	3	4	5
Patients, *n*	941	873	823	754	695
Age, years, (SD)	76 (10.3)	76.6 (10.3)	77.1 (10.2)	77.5 (10.2)	77.9 (10.1)
Female sex, *n* (%)	504 (53.6)	470 (53.8)	444 (53.9)	409 (54.2)	378 (54.4)
Systolic blood pressure, mmHg (SD)	133.6 (16.9)	132.5 (16.2)	133.4 (16.3)	133.6 (16.9)	135.6 (16.9)
Missing SBP data, *n* (%)	153 (16.3)	177 (20.3)	183 (22.2)	209 (27.7)	181 (26)
Diastolic blood pressure, mmHg (SD)	75.2 (11.9)	74.7 (11.2)	74.7 (10.8)	74.8 (10.8)	76 (10.8)
Missing DBP data, *n* (%)	155 (16.5)	179 (20.5)	187 (22.7)	210 (27.9)	184 (26.5)
LDL cholesterol, mmol/L (SD)	2.5 (0.9)	2.5 (0.8)	2.6 (1.0)	2.6 (0.9)	2.6 (1.0)
Missing LDL cholesterol data, *n* (%)	523 (55.6)	479 (54.9)	462 (56.1)	420 (55.7)	368 (52.9)
HbA1C, mmol/mol (SD)	53.3 (13.4)	52.7 (13.1)	53.3 (14.9)	53.1 (13.4)	52.8 (12.3)
Missing HbA1C data, *n* (%)	631 (67.1)	578 (66.2)	543 (66.0)	511 (67.8)	457 (65.8)
BMI (SD)	27.7 (4.5)	27.8 (4.6)	27.6 (4.6)	27.8 (4.9)	27.7 (4.6)
Missing BMI data, *n* (%)	512 (54.4)	448 (51.3)	425 (51.6)	397 (52.7)	391 (56.3)
Blood pressure <140/90 mmHg, *n* (%)	460 (58.4)	431 (62.1)	361 (56.6)	331 (60.8)	285 (55.7)
Blood pressure ⩽140/90 mmHg, *n* (%)	559 (71.0)	526 (75.8)	454 (71.2)	402 (73.9)	350 (68.4)
Antihypertensive treatment, *n* (%)	723 (76.8)	680 (77.9)	623 (75.7)	578 (76.7)	543 (78.1)
Antihypertensive drugs, *n* (SD)	1.4 (1.0)	1.4 (1.1)	1.4 (1.1)	1.4 (1.0)	1.4 (1.0)
LDL cholesterol < 2.6 mmol/L, *n* (%)	249 (59.6)	242 (61.4)	199 (55.1)	182 (54.5)	190 (58.1)
Lipid-lowering treatment, *n* (%)	556 (59.1)	502 (57.5)	432 (52.5)	408 (54.1)	378 (54.4)
Treatment with APT or OAC, *n* (%)	802 (85.2)	728 (83.4)	652 (79.2)	593 (78.6)	548 (78.8)
Smoker, *n* (%)	76 (12.1)	85 (12.4)	86 (12.3)	82 (12.3)	74 (11.6)
Missing smoking data, *n* (%)	313 (33.3)	187 (21.4)	126 (15.3)	85 (11.2)	57 (8.2)

SD: standard deviation; LDL: low density lipoprotein; HbA1c: glycated hemoglobin; APT: antiplatelet treatment; OAC: oral anticoagulation; SBP: systolic blood pressure; DBP: diastolic blood pressure; BMI: body mass index.

In logistic, mixed-effect regression models adjusted for sex and age, the first year of follow-up (from baseline to year 1) was associated with higher odds of achieving target levels of BP (odds ratio (OR) 1.60, 95% CI 1.34–1.92) and LDL-C (OR 2.33, 95% CI 1.76–3.07), (Table 3). There was no statistically significant association between smoking and follow-up the first year, but there was a significant association between the first year of follow-up and higher odds of concomitantly attaining target BP, LDL-C and non-smoking status (OR 2.08, 95% CI 1.38–3.13). In contrast, there was no significant association between change in risk-factor control and follow-up per year from year 1 to year 5.

**Table 3.** Association between change in risk-factor control and secondary prevention treatment per year of follow-up in logistic mixed-effect regression analysis.

		Odds ratio	95% CI	*p* Value
*Risk-factor control*				
BP <140/90 mmHg	Baseline to year 1	1.60	1.34–1.92	<0.001
	Year 1 to year 5	0.98	0.93–1.03	0.37
LDL-C < 2.6 mmol/L	Baseline to year 1	2.33	1.76–3.07	<0.001
	Year 1 to year 5	0.94	0.89–1.00	0.05
Non-smoker	Baseline to year 1	1.10	0.97–1.25	0.22
	Year 1 to year 5	0.94	0.90–0.99	0.01
All three	Baseline to year 1	2.08	1.38–3.13	<0.001
	Year 1 to year 5	0.93	0.87–1.00	0.06
*Secondary prevention treatment*				
Antihypertensive treatment	Baseline to year 1	1.23	1.05–1.44	0.01
	Year 1 to year 5	0.97	0.93–1.01	0.16
APT or OAC treatment	Baseline to year 1	8.19	6.53–10.27	<0.001
	Year 1 to year 5	0.85	0.81–0.89	<0.001
Statin treatment	Baseline to year 1	3.41	2.90–4.02	<0.001
	Year 1 to year 5	0.91	0.88–0.95	<0.001
All three	Baseline to year 1	4.69	3.87–5.68	<0.001
	Year 1 to year 5	0.94	0.90–0.98	0.002

BP: blood pressure; LDL-C: low-density lipoprotein cholesterol; APT: antiplatelet treatment; OAC: oral anticoagulation; CI: confidence interval.

### Secondary prevention treatment

**Figure 3.**
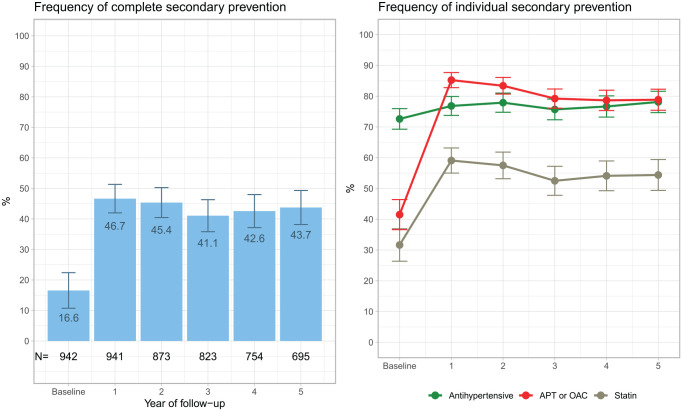
Relative frequency of use of three (complete) secondary prevention treatments(left) and individual secondary prevention treatment(right) after a first transient ischemic attack. The black numbers at the bottom of the left plot show the absolute number (N) of patients for whom complete drug-dispensing data were available. APT: antiplatelet treatment; OAC: oral anticoagulation.

The use of antihypertensive treatment increased numerically during follow-up (Figure 3), compared to baseline, and usage was highest in the fifth year of follow-up at 78.1%. Both APT/OAC and statin treatment increased in frequency after baseline and was highest in the first year, 85.2% and 59.1%, respectively.

In regression models, the first year of follow-up (from baseline to year 1) was associated with higher odds of antihypertensive (OR 1.23, 95% CI 1.05–1.44), APT/OAC (OR 8.19, 95% CI 6.53–10.27) and statin (OR 3.41, 95% CI 2.90–4.02) treatment (Table 3). Conversely, follow-up during years 1 through 5 was associated with lower odds of APT/OAC (OR 0.85, 95% CI 0.81–0.89) and statin (OR 0.91, 95% CI 0.88–0.95) treatment per annum.

## Discussion

### Main findings

This registry-based longitudinal observational study from primary care of 942 patients with a first TIA showed that control of BP and LDL-C improved in the first year of follow-up but not subsequently. Only about a third of patients concomitantly attained a BP <140/90 mmHg and an LDL cholesterol < 2.6 mmol/L and were non-smokers at some point after their first TIA. Secondary prevention treatment increased in the first year after a TIA, but APT/OAC and statin treatment then decreased slightly, despite inadequate risk-factor control. Overall, less than half of the patients received treatment with all three of antihypertensives, statins and APT/OAC after a first TIA.

### Other findings and comparisons to other studies

After a TIA or minor stroke, 13% of patients experience another major cardiovascular event within 5 years, with about half the events occurring during the first year.^1^ Accordingly, there is a pressing need to achieve target levels for all risk factors, in particular with respect to blood pressure.^4,5^ Although more than 75% of the patients in our study used antihypertensive treatment, less than 60% attained BP <140/90 mmHg during follow-up, which exposes them to an increased, yet avoidable risk of new cardiovascular disease. On average, patients used just 1.4 antihypertensive drugs per person, which along with inadequate control suggests considerable room for improvement. By comparison, the treatment group of the older subcohort of the SPRINT study used 2.6 antihypertensive drugs per person.^21^ Control of BP was lower at 1-year follow-up in our data when compared to a Canadian study from a university hospital (58%vs 86%), though that study featured younger patients (76vs 65 years), but similar to an Irish study from a primary care setting (58%vs 64%).^9,10^ A Scottish study has shown that the prescription of antihypertensive treatment to patients after a hemorrhagic stroke can be augmented through a quality improvement program.^22^ Similar initiatives may be beneficial in a primary-care setting too and might contribute to improved blood pressure control.

Statin use was surprisingly low in our data, never reaching 60% at any point during follow-up, despite being recommended for patients after a TIA, regardless of LDL-C level.^6^ The low use was reflected in LDL-C control, which was attained in only about 60%. Even lower statin use was reported in a large Italian follow-up study of 7 776 patients with TIA, in which merely 34% received lipid-lowering treatment 1 year after hospital discharge.^11^ In the same study – which did not feature risk-factor data on BP, LDL-C and smoking – patients who did use lipid-lowering treatment had a 28% lower risk of the composite outcome of all-cause death, and hospitalization for stroke or myocardial infarction. Statin use was higher in a Norwegian study (76%) and the previously cited Irish study (75%), which both comprised patients with stroke or TIA who were managed in a primary-care setting.^9,23^

The use of APT/OAC at year 1 of follow-up in our data was similar to that of the large Italian study of patients with TIA mentioned previously (85%vs 89%).^11^ In their work, they also showed that treatment with APT/OAC was associated with a 27% lower risk of all-cause death, and hospitalization for stroke or myocardial infarction. In our study, APT/OAC use decreased through years 2–5 of follow-up.

There is documented uncertainty in primary care on how secondary prevention treatment should be utilized, especially in older patients, such as those who have had a TIA.^24^ This suggests that clearer recommendations on risk factor-goals from hospital-based physicians in discharge notes and referrals may aid in target attainment.

Finally, statistically significant improvement in risk factor-control and secondary prevention treatment was only observed in the first year of follow-up after a first TIA, but not in years 1 through 5. This suggests that the observed, early improvements in risk factor-control are the result of treatment changes during the immediate aftermath of the TIA. After the first year of follow-up, risk factor-control stagnated as secondary prevention treatment declined.

### Strengths and limitations

This study has several strengths: Through QregPV, all patients visiting any primary-care center in the Region of Västra Götaland were included in a systematic and unbiased manner based on the presence of any of the common diagnoses of hypertension, ischemic heart disease, diabetes mellitus, asthma or chronic, obstructive pulmonary disease. QregPV also provides longitudinal risk-factor data collected in routine care. Via linkage to national and regional registers, we also had access to high-quality data on concomitant diseases and drug dispensations.

The study also has important limitations: Because variables are collected from routine practice, not all variables were assessed every year for every patient, which lead to a high frequency of missing data, especially for LDL cholesterol and smoking. This limits the internal validity of the study. We also acknowledge that only patients with a TIA who visited primary care and had a qualifying diagnosis – such as hypertension, ischemic heart disease or diabetes mellitus – that resulted in inclusion in QregPV were included in the study. This is manifested in the high prevalence of patients with hypertension, ischemic heart disease and diabetes mellitus in our material. In the Region of Västra Götaland, patients who have had a TIA are generally offered one follow-up visit 3 months after hospital discharge and are then referred to primary care. Some of the initial follow-up visits occur as hospital-based, outpatients visits in clinics specializing in stroke, internal medicine, or geriatrics, whereas other follow-up visits occur in primary care. Some patients with severe comorbidities, such as advanced heart failure or type 1 diabetes mellitus, may be subject to hospital-based outpatient follow-up solely with their cardiologist or diabetologist, respectively. If these patients were not followed in primary care for any other diagnosis, such as hypertension or asthma, they might not be encompassed by this study. Finally, study inclusion was based on a primary hospital discharge diagnosis of TIA, which is a less accurate diagnosis than stroke.^13,14^ We elected to only include patients discharged from stroke units, internal medicine, neurology or geriatrics to increase the specificity of the TIA diagnosis. This may itself also constitute a limitation, since patients managed at other wards were not included in the study.

## Conclusion

There is considerable room for improvement in risk-factor control and secondary prevention treatment in patients with a first TIA who are followed in primary care in Sweden. A more structured approach with initiation of adequate medications for risk-factor control already in the in-hospital setting, and improved follow-up in primary care beyond the first year after a TIA appears to be desirable and would likely contribute to increased secondary prevention treatment, subsequent improved risk factor-control and – ultimately – decreased risk of cardiovascular disease recurrence.
